# Correction: Comparison of frailty instruments for predicting mortality and prolon ged hospitalization in acute coronary syndrome patients

**DOI:** 10.1371/journal.pone.0339683

**Published:** 2025-12-23

**Authors:** Anne Langsted, Jocelyne Benatar, Andrew Kerr, Katherine Bloomfield, Gerry Devlin, Alexander Sasse, David Smythe, Andrew To, Harvey White, Gerrard Wilkins, Ralph Stewart

The word “prolonged” is misspelled in the article title. The correct title is: Comparison of frailty instruments for predicting mortality and prolonged hospitalization in acute coronary syndrome patients. The correct citation is: Langsted A, Benatar J, Kerr A, Bloomfield K, Devlin G, Sasse A, et al. (2025) Comparison of frailty instruments for predicting mortality and prolonged hospitalization in acute coronary syndrome patients. PLoS ONE 20(2): e0318656. https://doi.org/10.1371/journal.pone.0318656.

In Fig 3, the word “patients” in the heading is misspelled. Please see the correct Fig 3 here.

**Fig 3:**
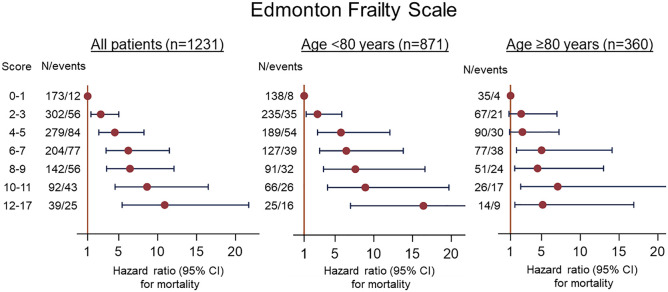
Associations between Edmonton frailty scale score and mortality for all patients and patients aged <80 years and ≥80 years. The EFS is reported for the range of scores including patients who do not meet criteria for mild or greater frailty (EFS score ≤5).
