# Supplementary material for: Comparison of frailty instruments for predicting mortality and prolon ged hospitalization in acute coronary syndrome patients
Source: PLoS One. 2025 Feb 7;20(2):e0318656. doi: 10.1371/journal.pone.0318656 (PMC11805369; doi:10.1371/journal.pone.0318656)
Supplement: Attachment — Submitted filename: PLOS one reply to reviewers v4.docx [file pone.0318656.s001.docx]

We are grateful to the reviewers for their thoughtful comments and suggestions.

In response we have undertaken additional analyses and made changes to the manuscript as suggested. Our replies to reviewer comments and changes made to the manuscript are listed below.

**Reviewer #1:**

- From a methodological viewpoint, the inclusion of the GPCog cognitive test may not be necessary. Limiting the comparison to frailty assessment tools alone would make the study more streamlined and interpretable.
- The Katz index is primarily a measure of disability, which is a distinct phenomenon than frailty. The ADL levels are not highly discriminative in the studied population, and it is unclear whether the tables reference lost or maintained ADLs. So, it can be omitted from the analysis.

Reply: The GP Cog and Katz scores are now not included.

Reviewer #1:

In Figure 1, for the calculation of Cox regression for the EFS, I would like to see the following scoring: 0-5 = Not Frail; 6-7 = Vulnerable 8-9 = Mild Frailty; 10-11 = Moderate Frailty; 12-17 = Severe Frailty.

Reply: The scoring of frailty using the EFS has been changed in figure 1 as suggested, and revised the methods and results sections accordingly.

An additional observation was a graded increase in mortality and prolonged hospitalisation risk with increase for patients with an EFS score <=5, who do not meet these criteria for frailty. We have illustrated this in an additional figure 3, which also displays results stratified by age <80 and >=80, as suggested by reviewer 2.

Reviewer #1:

It should be noted that none of the frailty assessment tools used were specifically developed for acute settings, and there is a lack of evidence regarding their reproducibility in this context. This limitation should be explicitly mentioned.

Reply:

We agree with this limitation.

See discussion, study limitations. “Assessments undertaken during an acute medical admission may have been less reliable indicators of usual status.”

Reviewer #1:

Although the methods state that the evaluations were conducted when patients were clinically stable, it is important to clarify whether the Clinical Frailty Scale refers to the level of frailty before the acute coronary syndrome event or at the time of the ACS. Given that the CFS reflects overall clinical judgment, there is a potential for overestimating frailty in patients assessed during this acute phase.

Reply: See Methods section under ‘frailty instruments’ and limiyations section as above

“Frailty assessments were undertaken by a trained research nurse >24 hours after admission and when the patient was clinically stable, after obtaining written informed consent. Response to questions considered the patient’s status during the 2 weeks before the acute illness leading to hospitalization…”

Reviewer #1:

In the discussion, it is essential to highlight the multidimensional contribution of frailty to cardiovascular diseases. Please refer to and discuss the findings of Testa et al. (2020), which address physical versus multidimensional frailty in older adults with and without heart failure: "Testa G, Curcio F, Liguori I, Basile C, Papillo M, Tocchetti CG, Galizia G, Della-Morte D, Gargiulo G, Cacciatore F, Bonaduce D, Abete P. Physical vs. multidimensional frailty in older adults with and without heart failure. ESC Heart Fail. 2020 Jun;7(3):1371-1380. doi: 10.1002/ehf2.12688. Epub 2020 Apr 3. PMID: 32243099; PMCID: PMC7261566."

Reply: Reference to the study by Testa (reference 29) has been included. See discussion section

Under associations between frailty scores and outcomes.

“The EFS is a multidimensional assessment which includes evaluation of physical activity, weight loss, activities of daily living, cognitive function and social support (12). ……….. In a study of patients hospitalized with heart failure a multi-dimensional frailty instrument also had better risk discrimination compared to a physical frailty score (29) “

And Under comparison with other studies

**“**Previous studies have reported associations between frailty assessed using the Fried criteria (22, 23),, the CFS (24, 25, 26), the EFS (27, 28), as well as other frailty instruments (29) and all cause mortality and hospitalisations.

**Reviewer #2:**

I have some suggestions. ADL are measure of disability and I think not appropriate to use Kats index as measure of frailty, similarly GPCog measure one of the frailty domain.

Reply:

We agree the Katz and GP Cog are not measures of frailty, and have removed these from the manuscript.

Reviewer #2:

The AUC found in your analysis could also be evaluating stratifying the sample by age (i.e. 70-80 and > 80). It should be of interest to evaluate the results of a cox regression analysis on your outcome to verify the effect of the different instruments of frailty.

Reply:

We have undertaken an additional analysis stratified by age <80 years and >= 80 years as suggested. C-statistics are reported in table 5. This analysis demonstrated that comparisons of frailty tools were consistent across age groups, and that risk discrimination appears to be better for patients aged <80 years. Results of Cox regression analyses are displayed for EFS in the new figure 3.

Reviewer #2:

Why measure in this setting Euroscore II?

Reply:

The reasons for including adjustment for Euroscore II are now more clearly documented.

See methods section

“The Euroscore II estimates 30 day mortality after cardiac surgery (17). Euroscore has also been associated with mortality in coronary heart disease patients treated by percutaneous coronary stenting (18) and with mortality during ten years follow up (19). ……”

And discussion under ‘clinical implications’

“ ….. For example, in patients with ischemic left ventricular dysfunction the benefits of coronary artery bypass surgery compared to medical therapy are less in older patients (31) and those with a lower exercise capacity (32). In the current study coronary artery bypass surgery was performed in <10% of patients who were ‘moderately frailty’ compared to 27% graded as ‘very fit’, The Euroscore is commonly used to estimate 30 day mortality risk after cardiac surgery (19), and predicts mortality after percutaneously coronary intervention (18), and long term mortality (19), The observation that each of the frailty instruments improved risk discrimination when added to Euroscore II supports including evaluation of frailty for patients considered for cardiac surgery.

Changes to tables and figures.

Tables 2 to 4 have been revised by removing data on Katz and GPcog scores.

The new table 4 combines relevant data from 2 previous tables.

The new table 5 reports C-statistics for each of the frailty tools for mortality stratified for ages <80 years and >=80 years.

Figures 1 and 2 have been revised by removing HR plots for CGCog and Katz scoers.

The new Figure 3 reports HR for mortality for the EFS in qppoximate quintiles of the study population, and stratified by age <80 years and >=80 years.

A manuscript with track changes is attached. Changes made to the text are underlined. Information removed for the text and tables is not included in these track changes.

Thank you for your considering this revised manuscript.

Ralph Stewart.
